# Street Food and Takeaway Food Purchasing Patterns in Bosnia and Herzegovina

**DOI:** 10.3390/ijerph19159086

**Published:** 2022-07-26

**Authors:** Sofia Sousa, Inês Lança de Morais, Gabriela Albuquerque, Marcello Gelormini, Aida Filipović-Hadžiomeragić, Dragana Stojisavljevic, Albertino Damasceno, Pedro Moreira, João Breda, Nuno Lunet, Patrícia Padrão

**Affiliations:** 1 EPIUnit—Instituto de Saúde Pública, Universidade do Porto, Rua das Taipas 135, 4050-600 Porto, Portugal; sofia.sousa@ispup.up.pt (S.S.); gabriela.albuquerque@ispup.up.pt (G.A.); tino_7117@hotmail.com (A.D.); pedromoreira@fcna.up.pt (P.M.); nlunet@med.up.pt (N.L.); 2 Laboratório para a Investigação Integrativa e Translacional em Saúde Populacional (ITR), Rua das Taipas 135, 4050-600 Porto, Portugal; 3 Faculdade de Ciências da Nutrição e Alimentação da Universidade do Porto, Rua do Campo Alegre 823, 4150-180 Porto, Portugal; 4 Nutrition, Physical Activity and Obesity Programme, Division of Noncommunicable Diseases and Life-Course, World Health Organization (WHO) Regional Office for Europe, UN City, Marmorvej 51, 2100 Copenhagen, Denmark; inesbolm@gmail.com (I.L.d.M.); marcello.gelormini@gmail.com (M.G.); 5 Public Health Institute of the Federation of Bosnia and Herzegovina, Tahtali Sokak 17, 71000 Sarajevo, Bosnia and Herzegovina; a.filipovic@zzjzfbih.ba; 6 Public Health Institute of the Republika Srpska, Jovana Dučića 1, 78000 Banja Luka, Bosnia and Herzegovina; dragana.stojisavljevic@med.unibl.org; 7 Faculty of Medicine, University of Banja Luka, 14 Save Mrkalja, 78000 Banja Luka, Bosnia and Herzegovina; 8 Departamento de Ciências da Saúde Pública e Forenses e Educação Médica, Faculdade de Medicina da Universidade do Porto, Alameda Prof. Hernâni Monteiro, 4200-319 Porto, Portugal; 9 Faculdade de Medicina, Universidade Eduardo Mondlane, Avenida Salvador Allende 702, Maputo 1100, Mozambique; 10 Centro de Investigação em Atividade Física, Saúde e Lazer, Universidade do Porto, Rua Dr. Plácido Costa, 91, 4200-450 Porto, Portugal; 11 WHO Regional Office for Europe, 10675 Athens, Greece; rodriguesdasilvabred@who.int

**Keywords:** street food, takeaway food, ready-to-eat food, purchasing patterns, food choice, Eastern Europe

## Abstract

This study aimed to describe street food and takeaway food purchasing patterns in Sarajevo and Banja Luka, throughout the day and by city location. A cross-sectional evaluation of street food and takeaway food customers was conducted in 2017. All eligible vending sites (n = 348) in the vicinity of selected markets and bus stops were included. Data on the food items purchased, and time and geographic location of the purchases were collected. A total of 755 customers purchased 929 food items. Takeaway venues showed higher customer influx (5.0 vs. 2.0 customers observed per 10 min of observation, *p* < 0.001) and buying rates (6.7 vs. 2.0 items bought per 10 min of observation, *p* < 0.001; 1.5 vs. 1.0 items bought per customer, *p* < 0.001) than street food sites. These rates were higher in city peripheries for street food venues, and in city centres for takeaway establishments. The purchase of industrial food products prevailed throughout the day in street food venues, whereas most takeaway purchases comprised homemade foods, with or without industrial beverages. The proportion of customers buying foods and beverages together was higher in takeaway venues (15.3% vs. 6.0%, *p* < 0.001), especially during lunchtime and in city centres. In street food vending sites, sweet and savoury snacks seemed to be preferred in the afternoon, whereas in takeaway food establishments, savoury pastries and main dishes were mostly purchased at breakfast or lunch, and bread during the morning. Soft drinks and industrial juices were frequently purchased in both types of vending site and at all hours of the day, particularly in the afternoon. Our findings provide an overview of street food and takeaway food buying habits and consumer demands in these cities, reflecting local food culture and dietary behaviours. The identification of the meal contexts and city sub-regions in which specific purchasing practices emerge point to potential priority targets. These insights can be useful when designing interventions adapted to the specificities of these food environments and the food habits of customers.

## 1. Introduction

Following the collapse of communist regimes at the end of the 20th century, a set of socioeconomic transformations, including rapid industrialization, urbanization and economic development, occurred in Eastern European countries [1]. In Bosnia and Herzegovina, during the last 10 years the national gross domestic product increased by 19.8% [2] and the proportion of urban dwellers grew from 46% to 49% [3].

Rooted in these demographic and economic transitions, several changes in lifestyle and dietary habits were observed, especially in urban centres [1], including increasingly sedentary behaviours [4,5] as well as a growing demand for foods prepared away from home [6,7]. In this context, ready-to-eat street-vended foods and beverages have an important role in the urban food environments of the Eastern European region [8,9,10]. Specifically in cities in Bosnia and Herzegovina, in addition to traditional street food vendors, takeaway food establishments, such as “Buregdžinicas” (places selling savoury pies), “Ćevabdžinicas” (places selling barbecue) and “Pekaras” (bakeries), are also key providers of street-vended meals [9,10].

Eating out-of-home has been associated with lower dietary quality [11], also being reported as a risk factor for overweight and obesity [11,12]. Recent data on street food and takeaway food environments in cities in Bosnia and Herzegovina showed a high availability of homemade food items (60.2% in Sarajevo and 56.8% in Banja Luka), but also a relevant number of venues selling foods and beverages produced by the food industry (41.6% in Sarajevo and 57.4% in Banja Luka). A very high availability of soft drinks and fruit juice-based beverages was also a common feature in these settings [9,10]. These results suggest a shift to more westernized eating habits among street food and takeaway food customers. Nonetheless, to the best of our knowledge, their purchasing patterns have not yet been studied.

Food environments have been described as “the interface where people interact with the wider food system to acquire and consume foods”. In this context, food acquisition has been identified as an important feature of food systems, interacting with food environments in a bi-directional way, as it both influences and is influenced by a complex network of external and personal drivers [13,14]. This vindicates the relevance of assessing food purchasing behaviours as significant targets for achieving healthier diets. However, although research on street food has grown in recent years, it focused mostly on its hygiene and safety, whereas its consumption or purchase were seldom explored [15]. Meanwhile, studies on takeaway food have been typically conducted in high-income countries, and frequently directed towards fast-food restaurants, while takeaway meals from small traditional outlets have been overlooked [16].

This study aimed to characterize street food and takeaway food purchasing patterns in the two main urban areas of Bosnia and Herzegovina (Sarajevo and Banja Luka), and to understand how these vary across time (throughout the day) and space (city centre vs. periphery).

## 2. Methods

### 2.1. Overview

This study was implemented as part of the FEEDCities (Food Environment Description in Cities) research project. The general methodology of this research project is published elsewhere [17]. For the purpose of this study, cross-sectional evaluations of street food and takeaway food customers were conducted between June and August 2017, in Sarajevo and Banja Luka, which are the two the largest and most urbanized cities in Bosnia and Herzegovina [18,19].

### 2.2. Eligibility Criteria

The Food and Agriculture Organization (FAO) and the World Health Organization (WHO) defined “street food” as “ready-to-eat foods and beverages prepared and/or sold by vendors or hawkers especially in the streets and other similar places” [20,21]. Similarly, a definition for “takeaway food vending site” was proposed by the United Kingdom authorities, as “an outlet whose primary business is the sale of ready-to-eat food and beverages for consumption off the premises” [22]. In general, street food vending units have a more informal setup (e.g., food stands, kiosks), whereas takeaway food vending sites are built within four permanent walls (Supplementary Figure S1). However, both types of business sell ready-to-eat food (homemade and/or industrial) which are bought to be consumed in similar contexts (i.e., in the streets, off the premises). As such, both these definitions were adopted in order to select the food vending sites eligible for this study.

Any customer approaching eligible street food or takeaway food vending sites during a pre-specified time period to buy ready-to-eat foods and/or beverages (hereafter referred to as “food items”) was eligible for the study. All age groups were considered as eligible.

### 2.3. Sampling Procedure

A preliminary assessment of the distribution of street food and takeaway food vending sites in each city was accomplished through field visits conducted in Sarajevo and Banja Luka, and taking into account information provided by the WHO country office and by the respective local authorities. Whereas in Sarajevo the vending sites were clustered in and around public markets, in Banja Luka these were mainly distributed in and around the main city market (Gradska tržnica), in addition to several public transportation stops (bus stations). As such, sampling procedures were adapted, taking into account these specificities, following a decision tree for the selection of vending sites, presented in the FEEDCities protocol [17].

In Sarajevo, 10 main public markets were selected in collaboration with local authorities, considering criteria related to the size and importance of the markets. Around the centre of the selected markets, a 500 m diameter buffer was defined, with the exception of the larger main city market (Markale), where a 1500 m diameter was needed to accommodate all the vending sites within its perimeter. In Banja Luka, the main city market was selected along with 10 bus stops, pre-selected as they corresponded to the main station of the city bus routes. To define the study area, a 1500 m buffer was drawn around the centroid of the main city market; and for each bus stop, the buffer diameter was 100 m. Within the study areas of each city, all publicly accessible streets were canvassed by pairs of field researchers, who identified all vending sites eligible for the study. All vending sites identified in each city were selected.

### 2.4. Data Collection

Data was collected by direct observation of each selected vending site, during which any customer buying one or more eligible food items, according to the abovementioned definitions, was assessed. The observation of each vending site ended when four customers were observed, or when a maximum period of 10 min was reached. Observations were performed in periods covering all days of the week and businesses’ working hours, from 8 a.m. to 6 p.m. A total of 348 vending sites were surveyed (224 in Sarajevo and 124 in Banja Luka), which corresponded to a total of 2941 min of observation.

Observations were performed by trained local field researchers, located at enough distance not to disturb the vendors’ regular activity or the customers’ usual behaviour. Training of field researchers occurred before data collection, and included theoretical, practical and field sessions, in order to standardize practices and maximize the quality of the collected data.

For each customer, two observers independently registered the food items purchased and respective quantities. Inter-observer concordance was evaluated using Cohen’s kappa (K) with 95% confidence interval (95%CI), being high to very high, regarding both variables (food item purchased: 98.2% agreement, K = 0.98, 95%CI 0.97–1.00; quantity purchased: 92.0% agreement, K = 0.89, 95%CI 0.86–0.92) [23]. Disagreements between observers were solved using a set of standardized criteria: (1) if the two observers registered different food items, the conflicting items were checked for their availability on the corresponding vending site (if only one of the conflicting items was available, that was the observation assumed, and both observations were disregarded if the conflicting items were both available or not available); (2) if the two observers registered the same food item, but with different degrees of specificity, the broadest observation was assumed; (3) if the two observers registered a different number of food items for the same customer, the observation with the larger number of food items available was assumed; and (4) if two observers registered the same food item, but different quantities, the average quantity of the two observations was assumed.

The purchased food items were grouped into sub-categories, based on the WHO nutrient profile model [24]: (1) main dishes; (2) bread; (3) savoury pastries; (4) savoury snacks; (5) sweet pastries and confectionery; (6) sandwiches; (7) coffee; (8) water; (9) soft drinks and industrial juices; (10) alcoholic beverages; and (11) yoghurt. Food items were also classified as homemade (foods and beverages that were prepared and/or cooked at home or in the street, even if using industrial ingredients) or industrial (foods and beverages that were produced by the food industry and sold as such, with no further preparation).

For each vending site observed, Global Positioning System (GPS) coordinates were recorded, as well as time of beginning and end of observation. Each selected buffer (market/bus stop) was classified as central or peripheral, according to their distance (below or above 2 km, respectively) to a city centre reference point, assigned in each city taking into account information provided by the respective local authorities and the WHO Country Office. The duration of the observation was also calculated for each vending site, as well as the customer influx (which was computed as the number of customers observed per 10 min of observation) and the food items buying rate (number of food items purchased per 10 min of observation). Purchases were then categorized taking into account time of the day, on an hourly basis (i.e., 8–9 a.m., 9–10 a.m., etc.) and city location (city centre vs. periphery).

### 2.5. Statistical Analysis

Absolute and relative frequencies were used to describe the food items purchased. Customer influx, food items buying rate and number of food items purchased per customer were described through median and percentiles 25 and 75. Pearson’s Chi-squared test was used to compare the street food purchases throughout the day and by city location. Mann–Whitney and Kruskal–Wallis tests were used to compare the customer influx, food items buying rate and number of food items purchased per customer, throughout the day or by city location, respectively. Differences were considered statistically significant when the critical level of significance (*p*) was less than 0.05. Statistical analyses were performed using *Stata*^®^ version 15.0 (StataCorp., College Station, TX, USA).

## 3. Results

**Figure 1:**
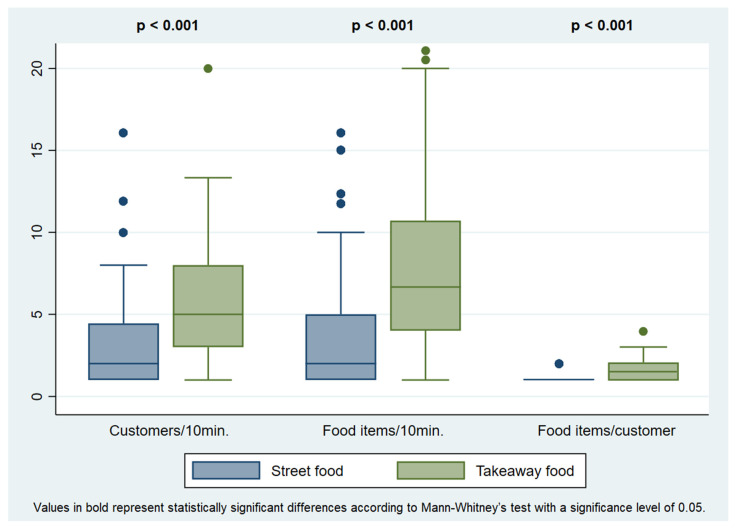
Customer influx, food items buying rate and number of food items purchased per customer, in the street food and takeaway food vending sites observed in Bosnia and Herzegovina.

A total of 755 customers purchased 929 food items. In each vending site, a median of three customers were observed over a median period of observation of 10 min. Takeaway food vending sites showed higher customer influx (5.0 vs. 2.0 customers/10 min, *p* < 0.001), food items buying rate (6.7 vs. 2.0 food items/10 min, *p* < 0.001) and number of food items purchased per customer (1.5 vs. 1.0, *p* < 0.001) when compared to street food vending sites (Figure 1). There were no significant differences between cities regarding these three variables.

**Figure 2:**
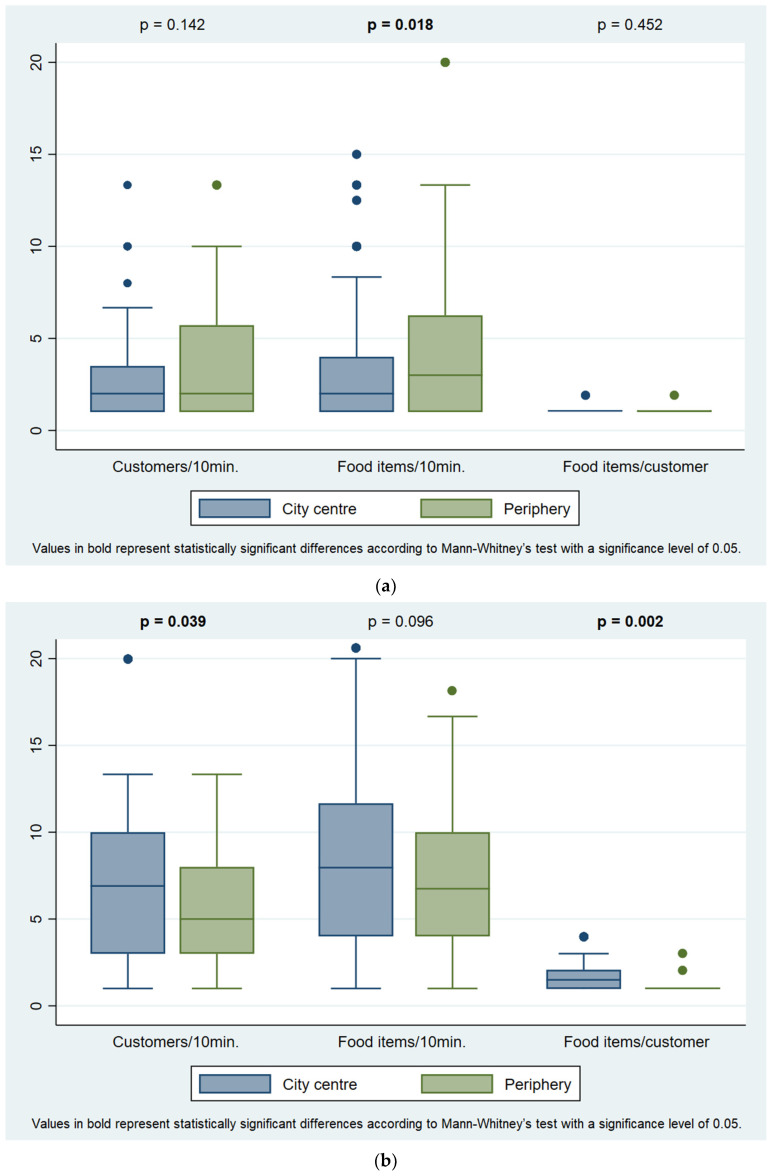
Customer influx, food items buying rate and number of food items purchased per customer, in (**a**) street food and (**b**) takeaway food vending sites, by city location.

Although no statistically significant differences were found, it was observed that customer influx and food items buying rate tended to be highest between 12 a.m. and 2 p.m. in street food vending sites, and from 10 a.m. to 1 p.m. in takeaway food vending sites (Supplementary Figure S2). Regarding city location, street food vending sites showed a higher food items buying rate in the periphery (3.0 vs. 2.0 food items/10 min, *p* = 0.018), whereas takeaway food vending sites presented higher customer influx (6.7 vs. 5.0 customers/10 min, *p* = 0.039) and number of food items purchased per customer (1.5 vs. 1.0, *p* = 0.002) in the city centre (Figure 2).

**Figure 3:**
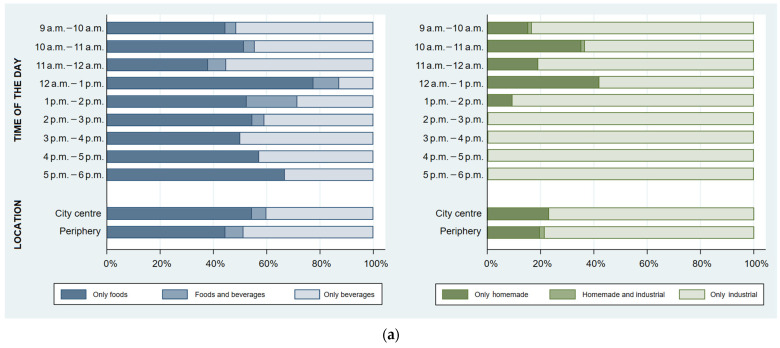

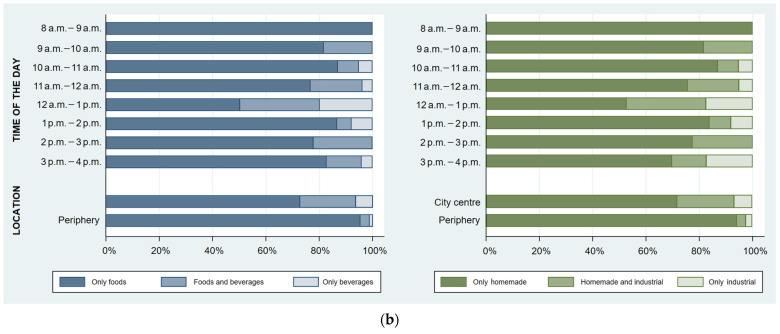
Proportion of customers purchasing foods and/or beverages and homemade and/or industrial food items, in the (**a**) street food and (**b**) takeaway food vending sites observed, throughout the day and by city location (street food: n = 298 purchases; takeaway: n = 457 purchases).

In street food vending sites, the purchase of foods was highest from 12 a.m. to 1 p.m. (87.1%) and from 1 p.m. to 2 p.m. (71.4%), whereas the purchase of beverages was higher during the morning period, especially between 11 a.m. and 12 a.m. (62.1%). Between 12 a.m. and 2 p.m., a greater frequency of joint purchase of foods and beverages was also observed. Customers buying industrial food items prevailed throughout the whole day, especially during the afternoon period. The purchase of homemade food items only occurred during the first half of the day, and was highest between 12 a.m. and 1 p.m. (41.9%). Regarding city location, no statistically significant differences were found in the purchase of foods and/or beverages, as well as homemade and/or industrial food items (Figure 3a).

Considering takeaway venues, the purchase of foods was very frequent (≥ 80%) throughout the whole day. Between 12 a.m. and 1 p.m., it was observed the highest frequency of the purchase of beverages (50.0%), as well as the joint purchase of foods and beverages (30.0%). The proportion of customers buying foods and beverages together was significantly higher in the city centre (20.8% vs. 3.4%, *p* < 0.001). The purchase of homemade and/or industrial food items followed similar distributions, since almost all foods purchased (99.6%) were homemade and almost all beverages bought (98.8%) were industrial (Figure 3b).

The joint purchase of foods and beverages was higher among takeaway food customers (15.3% vs. 6.0%, *p* < 0.001). In street food venues, 88.9% of joint purchases comprised both industrial foods and beverages (mostly sweet or savoury snacks plus soft drinks or juices), while in takeaway vending sites all these customers bought homemade foods with industrial beverages (mostly savoury pastries or main dishes with soft drinks, juices or yoghurt).

**Figure 4:**
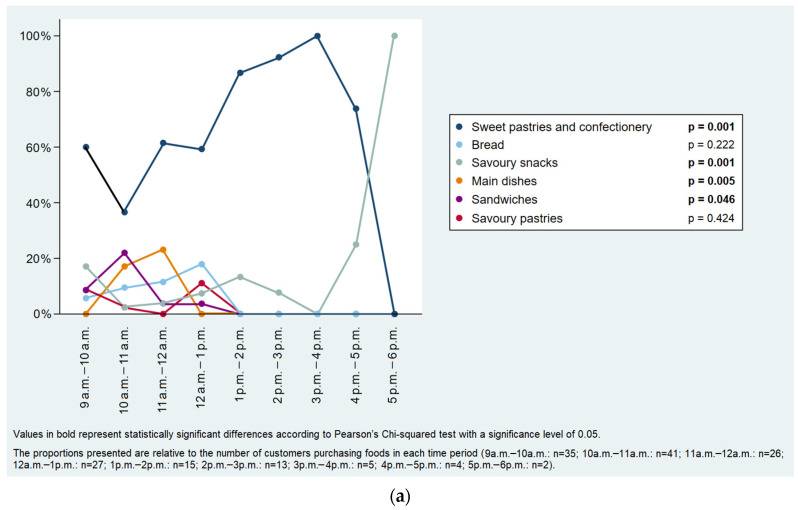

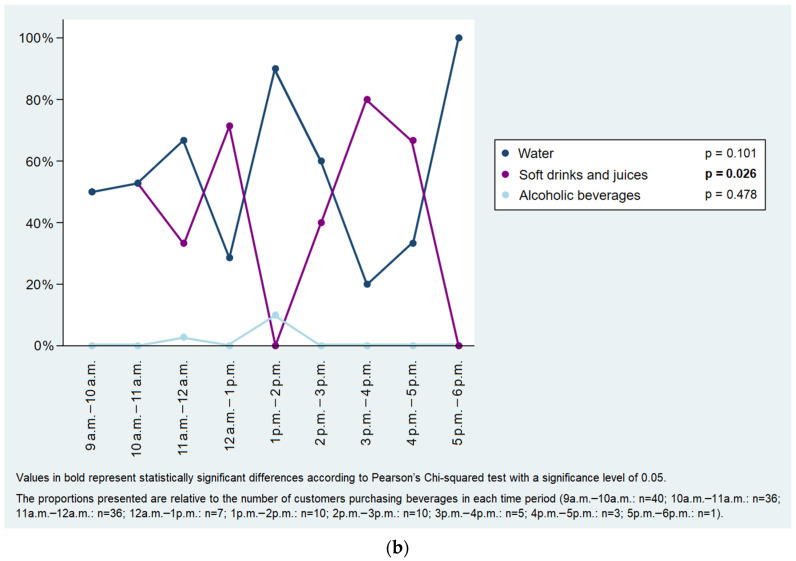
Distribution of the street food purchases throughout the day: (**a**) foods (n = 168) and (**b**) beverages. (n = 148).

**Figure 5:**
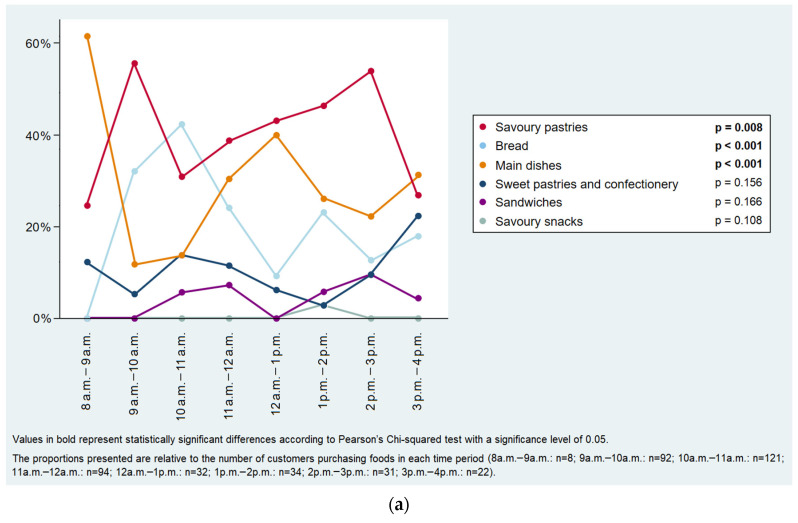

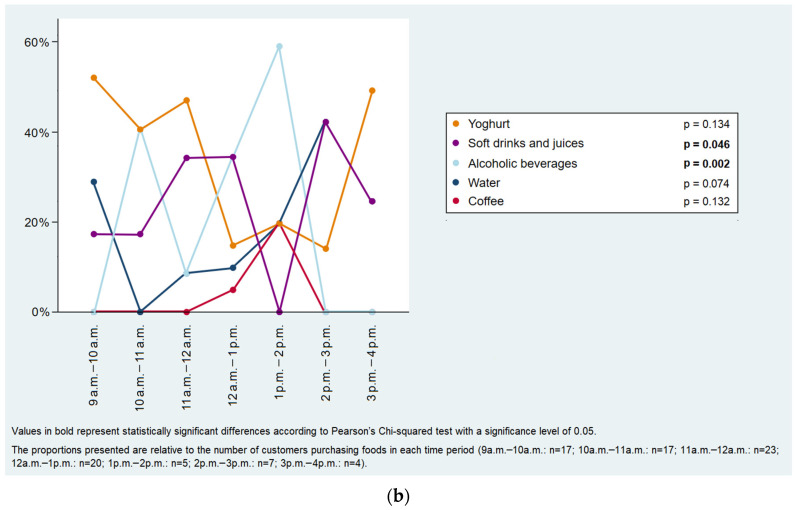
Distribution of the takeaway food purchases throughout the day: (**a**) foods (n = 434) and (**b**) beverages (n = 93).

In street food venues, among customers who bought foods, sweets pastries and confectionery were more frequently purchased between 1 p.m. and 5 p.m., savoury snacks between 4 p.m. and 6 p.m., main dishes from 10 a.m. to 12 a.m. and sandwiches from 10 a.m. to 11 a.m. (Figure 4a); among purchases including beverages, soft drinks and juices were most commonly bought between 3 p.m. and 5 p.m. and from 12 a.m. to 1 p.m. (Figure 4b). Regarding takeaway food purchases, savoury pastries were most frequently bought from 9 a.m. to 10 a.m. and from 2 p.m. to 3 p.m., bread between 9 a.m. and 11 a.m., main dishes between 8 a.m. and 9 a.m. and from 12 a.m. to 1 p.m. (Figure 5a), soft drinks and juices between 2 p.m. and 3 p.m., and alcoholic beverages from 1 p.m. to 2 p.m. (Figure 5b).

**Figure 6:**
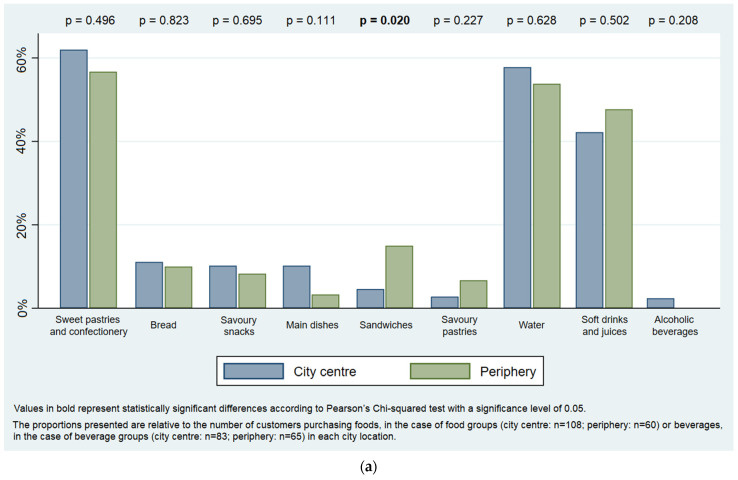

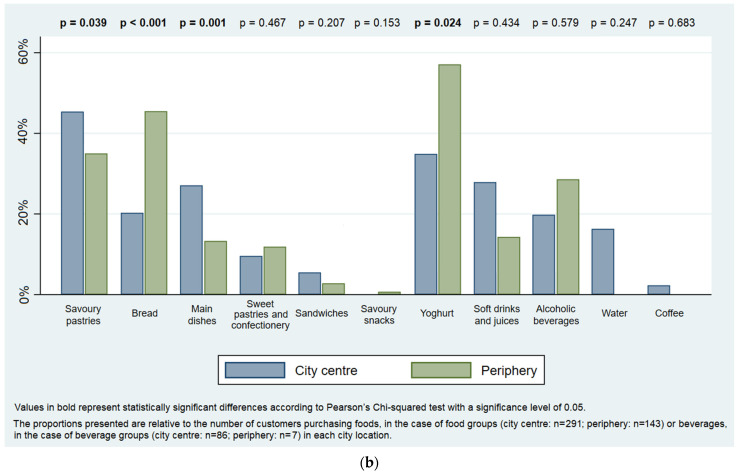
Distribution of (**a**) street food purchases and (**b**) takeaway food purchases, by city location.

Taking into account city location, among street food customers, the purchase of sandwiches was mostly observed in the peripheries (Figure 6a). In takeaway venues, the purchase of savoury pastries and main dishes was significantly more frequent in city centres, while bread was most commonly bought in the peripheries. Beverages were mostly bought in city centres, especially soft drinks and industrial juices, whereas the proportion of customers buying yoghurt was significantly higher in the peripheries (Figure 6b).

No statistically significant differences were found in the purchases made throughout the day and by city location, considering gender, age or weight status of the customers.

## 4. Discussion

Street food and takeaway food purchase in these two cities of Bosnia and Herzegovina was frequent, and included both homemade and industrially-produced food options. Customer influx and buying rates were higher in the city centres for takeaway food establishments, and in the periphery in the case of street food sites. The specific purchasing patterns found may reflect street food and takeaway food buying habits, which should be discussed in the context of the cultural and dietary specificities of these environments.

When compared to street food venues, takeaway food vending sites showed higher customer influx, food items buying rate and number of food items purchased per customer, indicating higher customers demand and a faster business activity. This is in line with the higher number of employees operating in takeaway establishments compared to street food vending sites (average number of employees: 6.1 vs. 3.2, unpublished data), which allowed for a faster service. Takeaway food activity was particularly faster in the city centres, whereas in street food vending sites, customer influx and purchasing rates tended to be higher in the periphery. This difference does not seem to be related to the geographical distribution of street food and takeaway vending sites, which was similar in both sub-regions [9,10], suggesting a higher demand for street food in the outskirts and for takeaway food in the centres of these cities. Considering the historical importance of street food in Eastern Europe [25], we hypothesize that, in cities from this region, buying in street food vending sites may still be a more culturally rooted habit, especially in the least modernized sub-areas, which could partly explain the higher demand for street food vending sites in peri-urban areas. On the other hand, takeaway food is a more recent phenomenon, which emerged with the growth and development of urban centres [16,26], where the concentration of people working in a variety of companies (e.g., industry, services) has been rapidly increasing. These consumers with limited time available for their meals may account for the greater demand for fast and home-like food options, which takeaway food vending sites can provide. The type of food items purchased also differed according to the type of vending site: in street food venues, customers purchased most frequently sweet pastries and confectionery, bread and savoury snacks, mostly industrially-produced, whereas in takeaway food establishments purchases were more directed towards homemade savoury pastries, bread and main dishes. This can be in part related to the physical setups encountered in these two venues. It was observed that takeaway food vending sites were more formal establishments, with better access to clean water and equipment that allows for the preparation and cooking of foods. On the contrary, street food venues were mostly small street-located kiosks also selling non-food products, such as newspapers and magazines, and were therefore less able to sell homemade products [9,10]. As a consequence of those differences, and as our results suggest, customers seem to turn to each of these types of vending sites under different circumstances. Whereas street foods seem to be more frequently bought in the context of a small snack, takeaway food may be more commonly consumed in the context of a more fulfilling home-like meal.

In street food venues, the purchase of foods, as well as the joint purchase of foods and beverages, reached their maximum values around lunchtime. These results were similar to those found in cities from Central Asia [27]. In contrast, beverages, mainly water and soft drinks/juices, were mostly acquired during the morning period. The purchase of industrial food products prevailed throughout the whole day in both the city centres and their outskirts, unlike cities from Central Asia where the most consumed street foods were homemade [28]. These findings suggest that the consumption of westernized foods and beverages may be a very common habit amongst Bosnian street food customers, in line with the growing availability and purchase of ultra-processed foods that has been observed in the region. In Central and Eastern Europe, sales of ultra-processed foods and drinks have been continuously increasing during the last two decades, reaching in 2019 values of 64 kg/capita and 77 L/capita, respectively [29,30]. This growth has been occurring as a consequence of the industrialization of food systems, technological development and globalization, especially in highly-populated and urbanizing middle-income countries [30], such as Bosnia and Herzegovina. On the other hand, the increasing customer demand for these types of foods may also have played a role in the development of food industries in the country, highlighting the importance of purchasing behaviours as modulators of food offer. This is one example of the complex two-way nature of the interactions among consumers, food environments and the whole food system [13,14]. This widespread consumption of ultra-processed food products is particularly concerning since it has been associated with several adverse health outcomes, including overweight, obesity, increased cardio-metabolic risk, cancer, type II diabetes, cardiovascular diseases, irritable bowel syndrome, depression, frailty conditions and all-cause mortality [31,32]. Sweet pastries and confectionery were the most frequently purchased street foods, which is in line with their high popularity among Bosnians [33,34]. These foods are not commonly consumed as a dessert in this country, but rather as snacks during the day or, in the case of sweet pastries, at main meals such as breakfast [33,35]. This is in line with our study, in which 40% of the sweet pastries purchased in street food venues were bought during the first hour of the day. Confectionery was mainly purchased during the afternoon period, similarly to other industrially produced items, such as savoury snacks, soft drinks and juices, indicating that these foods and beverages may be usually consumed by street food customers as afternoon snacks. Similar results were found in central Asian cities, where these food options were mostly bought as snacks in the mid-morning and/or mid-afternoon periods [27].

The purchase of homemade foods by street food customers was only observed during the first half of the day. This might be explained by the fact that most homemade foods sold in the context of street food were either prepared by vendors in their own homes or bought from another establishment [9,10], presumably at the beginning of each day. Between 12 a.m. and 1 p.m., almost half of the customers purchased homemade foods, mostly savoury pastries, such as “sirnica” (phyllo dough baked pie, filled with cheese) and “burek” (baked pie, filled with meat, usually beef), as well as bread, suggesting that these foods may be frequently consumed by street food customers at lunch. Although both bread and savoury pastries may usually be consumed at any time of the day, these foods are very commonly present in the context of a breakfast or lunch [33], which is in line with our results. Main dishes were mostly bought between 10 a.m. and 12 a.m., and 71.4% of these purchases corresponded to pizza, further suggesting the westernization of food habits among these customers.

In takeaway venues, the purchase of homemade cooked foods prevailed at all hours of the day, and in both city sub-regions, which is consistent with what was observed in central Asian street food environments [27], although contrasting with findings from similar settings in Mozambique and Moldova [36,37]. Drinks, mostly industrial, were rarely purchased alone, being mostly acquired together with foods. The joint purchase of foods and beverages was observed in approximately one in every six takeaway food customers, it being the highest between 12 a.m. and 1 p.m., and in city centres. Most of these meals were constituted by a savoury pastry, most frequently “burek” or “krompiruša” (meat or potato pie), or a main dish (mostly “ćevapi”), with a soft drink, a juice or a yoghurt, which is in line with what is usually consumed by locals outside the home at lunch. One of the most frequently consumed out-of-home foods is “ćevapi” (finger-sized barbecue-grilled ground meat, usually served with chopped onions in a split “lepinja”, a Bosnian yeast flat bread), as well as traditional pies with a range of savoury fillings [33,34]. Traditionally, yoghurt is another very important part of the Bosnian meals, especially in urban areas [33]. In addition to the lunchtime period, main dishes and savoury pastries were also most frequently purchased in the early morning, suggesting that these foods may also be habitually consumed by takeaway food customers in the context of a breakfast. As observed *in loco*, the Bosnian breakfast seems to usually be very fulfilling, it being common to find foods such as savoury or sweet pastries, or even main dishes, in this meal [35]. Our results indicate that traditional foods remain common options for these urban dwellers, suggesting that local gastronomic identity continues to be preserved in these food environments. However, high levels of trans-fat and sodium were found in the homemade foods sold in these urban environments, particularly savoury pastries and barbecue dishes [9,10]. Furthermore, industrially produced beverages, such as soft drinks and juices, were also very frequently bought, especially in the city centres, which may be a reflection of the nutrition transition that is taking place in the region [1].

Sandwiches were among the least frequently bought foods in both street food and takeaway food venues, despite being one of the most frequently available foods in both cities [9,10]. The same was found in central Asian street food customers [27]. The preparation of these type of foods at home by urban dwellers may be easier and quicker when compared to other street-vended options, which may partly explain the lower demand. The purchase of ready-to-eat fruit was also not observed in our sample, which can be explained by the low availability found (in Sarajevo and Banja Luka, 1.3% and 0.0% of all eligible vending sites sold ready-to-eat fruit, respectively) [9,10]. Vegetables were also rarely present in the products sold. Similar results were found in Central Asian cities [28], and reinforce concerns about the low consumption of fruits and vegetables in former Soviet Union countries [38]. The implementation of strategies focusing on the increasing availability and accessibility of these nutrient-dense foods has the potential to improve their consumption, that way leading to a healthier food environment, as documented in other settings [38,39].

### Strenghts and Limitations

Some strengths and limitations of the present study should be discussed. The observation efforts were distributed across all days of the week, which was an advantage for this work, since it allowed us to ensure that the variability over the week was covered when analysing purchase distributions throughout the day and by city location. One limitation was the fact that we assessed purchases, which may not correspond directly to consumption. However, foods or beverages which were not ready-to-eat were excluded from this study, in order to eliminate purchases intended for household consumption. Furthermore, all food items were acquired in single portions and mostly unpackaged or individually packaged, indicating that they should be mostly bought to consume immediately or shortly after. As such, these results are expected to provide an overview of the street food and takeaway food buying habits in these cities, which should indirectly reflect consumption habits. By identifying where and when specific patterns occur, we go deeper into the understanding of these customers’ practices and preferences, that way defining targets for action not only in terms of the meal context and city location, but also considering the type of vending site. Data collection was performed during summer months; as such, interpretation of these results should take into consideration the seasonality of these food environments. The implementation of a continuous monitoring system would contribute to overcome this limitation [40]. Direct observation of customers by trained researchers, instead of face-to-face interviews, was one of the strengths of the present work, because it allowed us to minimize interference with the vendors and their customers, which could lead to behavioural modifications and social approval bias [41]. Another strength that should be underlined was the high level of agreement between observers, which was a reflection of extensive training and constant supervision of the field researchers, leading to high quality observational data. Finally, the FEEDCities project is based on a standardized methodology [17], which allows for data comparability among different cities, countries or regions; however, due to local cultural specificities, the generalizability of our results is limited.

## 5. Conclusions

Purchases of street food and takeaway food in the two major urban areas of Bosnia and Herzegovina varied across time and space, reflecting specific patterns of purchase. Takeaway venues showed higher customer influx and buying rates in the city centres, whereas street food vending sites presented higher demand in the outskirts; and both types of establishments were busiest around lunchtime. Among street food customers, the purchase of industrial foods and beverages prevailed in both city centre and periphery, and throughout the day, whereas takeaway food customers purchased mostly homemade foods and industrial beverages. The patterns of street food and takeaway food purchase described in the present study contribute to better comprehend the buying habits of these customers. Further research focusing on the determinants of food choice by these consumers could give us additional insights on some of the patterns observed. This information can be useful in the development of public health policies more suitable to the cultural and dietary habits of the individuals who interact with these food environments. Such interventions (namely the improvement of food availability, educational strategies, nudging techniques, among others) could benefit from these insights as a reflection of customers’ behaviours, preferences and demands.

## Data Availability

The data presented in this study are available upon request to the corresponding author.

## Supplementary Materials

The following supporting information can be downloaded at: https://www.mdpi.com/article/10.3390/ijerph19159086/s1, Figure S1. Examples of (a) a street food vending site, and (b) a takeaway food vending site. Figure S2. Customer influx, food items buying rate and number of food items purchased per customer, in (a) street food and (b) takeaway food vending sites, throughout the day.
