# Supplementary material for: Street Food and Takeaway Food Purchasing Patterns in Bosnia and Herzegovina
Source: Int J Environ Res Public Health. 2022 Jul 26;19(15):9086. doi: 10.3390/ijerph19159086 (PMC9330047; doi:10.3390/ijerph19159086)
Supplement: Supplementary file 1 [file ijerph-19-09086-s001.zip › ijerph-1786188-supplementary.pdf]

**Supplementary Material**

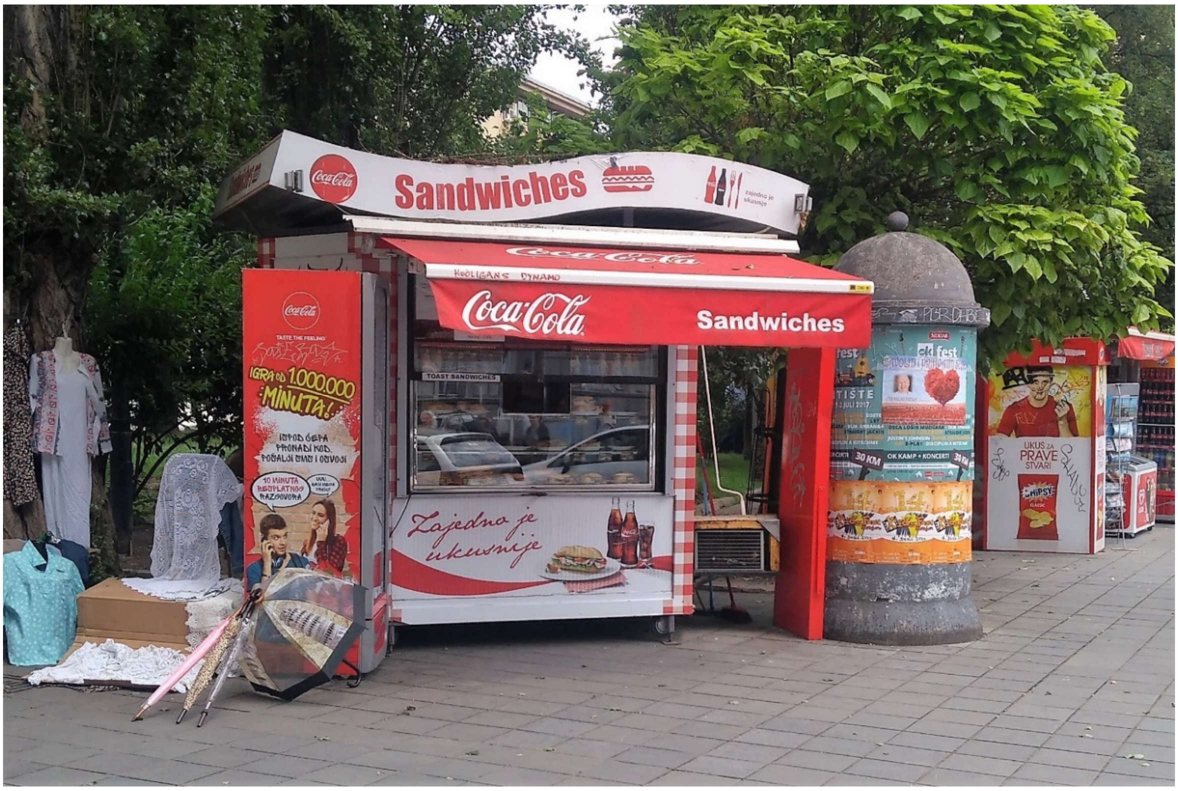


(**a**)

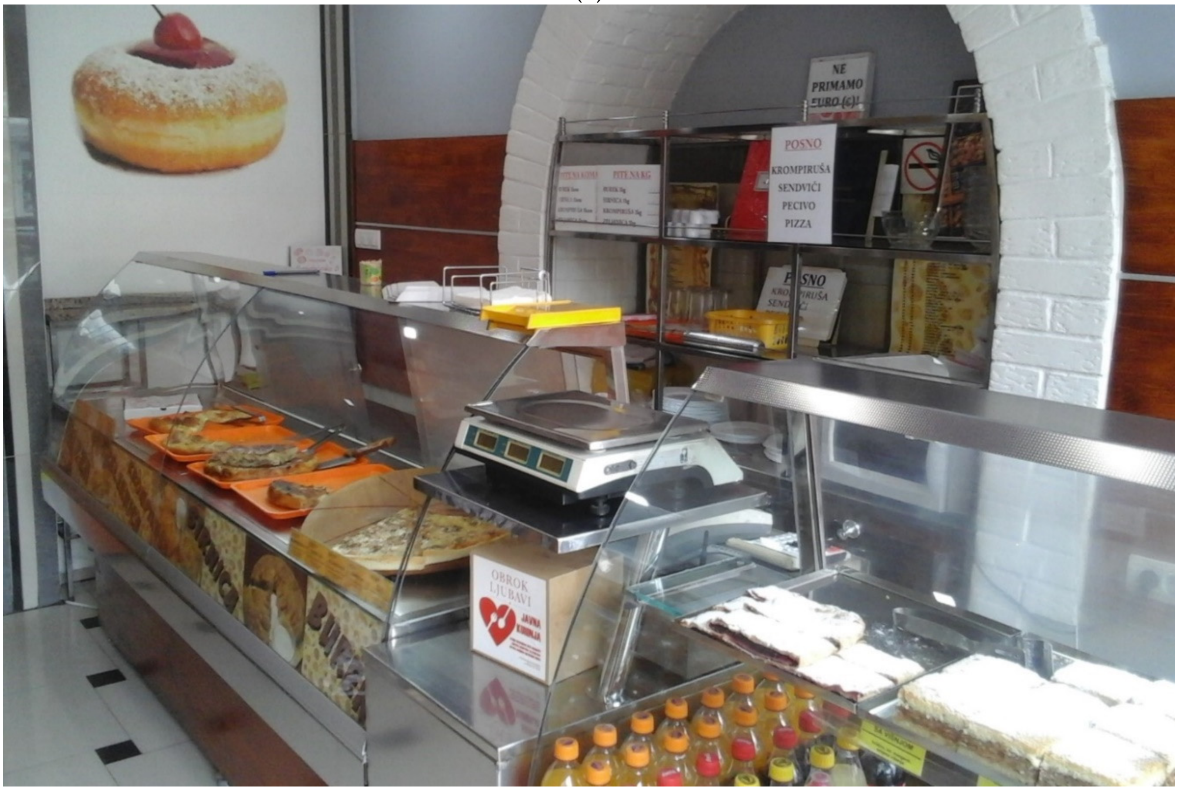


(**b**)

**Figure S1.** Examples of (**a**) a street food vending site, and (**b**) a takeaway food vending site.

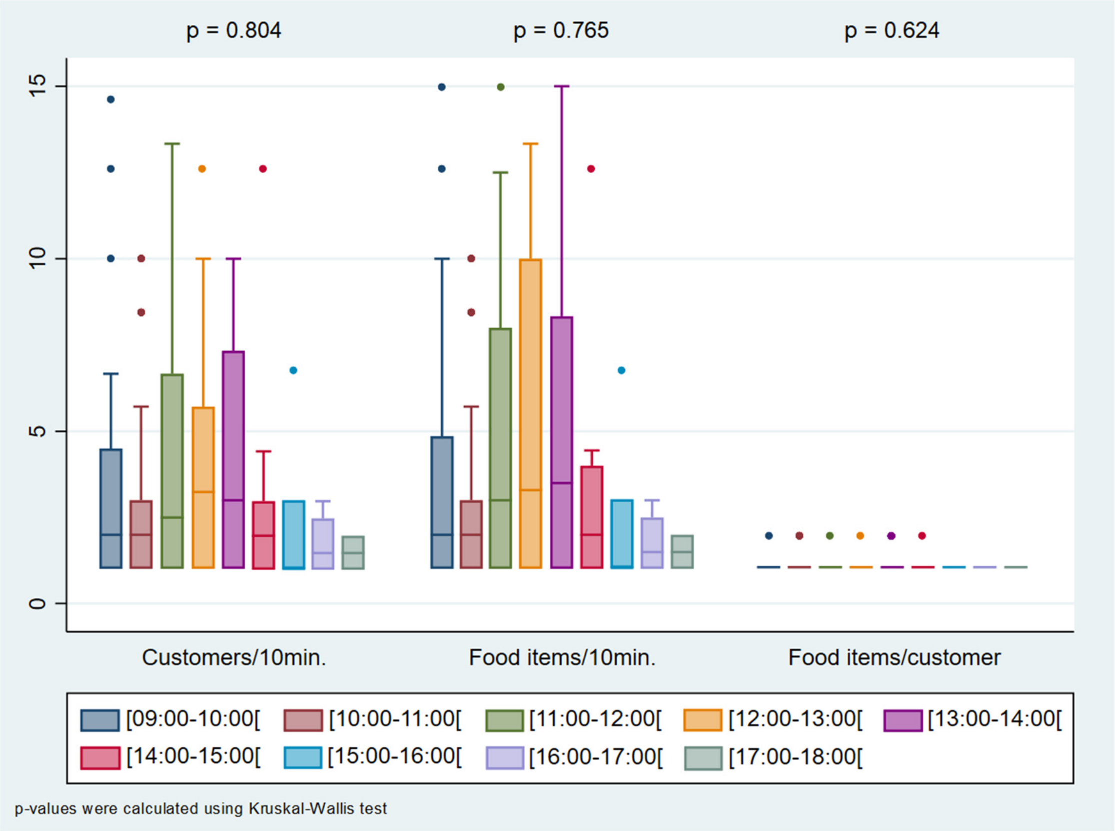


(**a**)

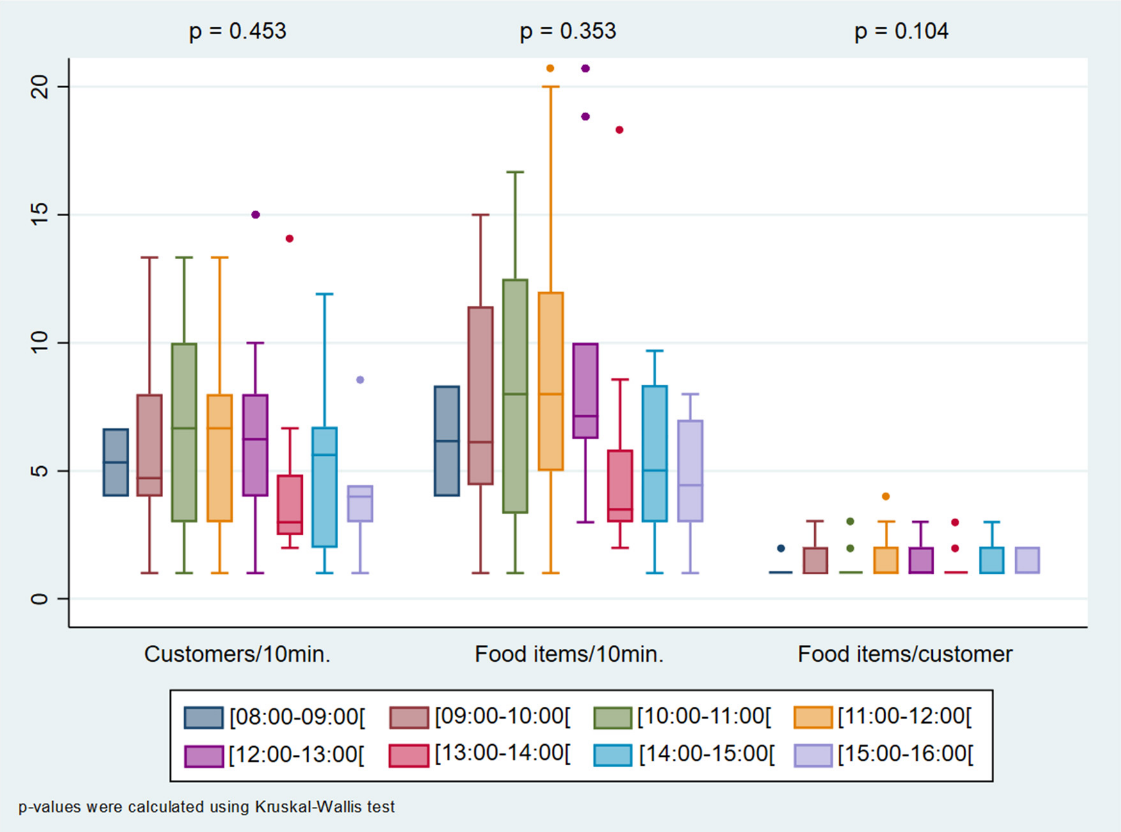


(**b**)

**Figure S2.** Customer influx, food items buying rate and number of food items purchased per customer, in (**a**) street food and (**b**) takeaway food vending sites, throughout the day.
